# Draft genome sequencing of a multidrug-resistant *Salmonella enterica* subspecies enterica serovar Typhimurium strain isolated from chicken in Bangladesh

**DOI:** 10.1128/mra.00619-23

**Published:** 2023-12-13

**Authors:** Najmun Nahar Popy, M. Nazmul Hoque, Mohammad Ferdousur Rahman Khan, Limon Biswas, Mohammad Habibur Rahman, Md. Saiduzzaman, Marzia Rahman, Md. Bahanur Rahman

**Affiliations:** 1 Department of Microbiology and Hygiene, Bangladesh Agricultural University, Mymensingh, Bangladesh; 2 Molecular Biology and Bioinformatics Laboratory, Department of Gynecology, Obstetrics and Reproductive Health, Bangabandhu Sheikh Mujibur Rahman Agricultural University (BSMRAU), Gazipur, Bangladesh; 3 Department of Neurology, Mymensingh Medical College Hospital, Mymensingh, Bangladesh; University of Maryland School of Medicine, Baltimore, Maryland, USA

**Keywords:** draft genome, multidrug resistance, *Salmonella enterica*, chicken, salmonellosis

## Abstract

Herein this study, we sequenced the genome of a multidrug-resistant *Salmonella enterica* serovar Typhimurium strain MBR-MFRK-23 isolated from the liver tissue of a diseased layer chicken. The 4,964,854-bp draft genome comprises 50 contigs with 50.5× coverage and 52.1% GC content and is typed as *S. enterica* sequence type 19.

## ANNOUNCEMENT

*Salmonella* is one of the most important foodborne zoonotic pathogens, and becoming multidrug resistant poses an imminent hazard to global public health (1). Salmonellosis, caused by *Salmonella* spp., is one of the most common infectious diseases in the poultry industry worldwide (2, 3). In poultry, *S. enterica* serovar Typhimurium causes infection in newborn chickens (4).

*S. enterica* serovar Typhimurium MBR-MFRK-23 strain was isolated from liver tissue of diseased layer chicken, collected from the poultry farms of Mymensingh district (24.7539° N, 90.4073° E) of Bangladesh in 02 April 2022. The MBR-MFRK-23 strain was identified according to ISO 6579-1: 2017 method for identification and serotyping, respectively (5). Sliced-up tissue was pre-enriched in buffered peptone water (BPW) at 1:9 ratio and incubated for 12 h at 37°C. Thereafter, 0.1 mL BPW suspension was then added to 10 mL of Rappaport-Vassiliadis soya peptone broth for 24 h at 42°C. A loopful of broth culture was streaked over Salmonella-Shigella agar (SS agar, HiMedia, India) and incubated for 24 h at 37°C (ISO 6579, 2017). Confirmatory identification of the *Salmonella enterica* species was done by PCR targeting *bcfC* gene (6). Serovar-specific identification of *S*. Typhimurium was done by *fliC* (7) gene-based PCR. Antimicrobial susceptibility patterns of the isolates were tested using the Kirby-Bauer disk-diffusion method, according to the guideline of the clinical and laboratory standards institute (8). *S.* Typhimurium strain MBR-MFRK-23 was resistant to sulfonamide, sulfamethoxazole, streptomycin, and cefotaxime. Pure colony (from SS agar plate) of the MBR-MFRK-23 isolate was cultured overnight on nutrient agar at 37°C for genomic DNA extraction from a single pure colony using QIAamp DNA Mini Kit (QIAGEN, Hilden, Germany) (9, 10), following the manufacturer’s instructions (Qiagen). The quality and concentration of the genomic DNA were checked using NanoDrop 2000 UV-Vis Spectrophotometer (Thermo Fisher, Waltham, MA, USA). Draft-genome sequencing of the MBR-MFRK-23 isolate was performed through Illumina MiSeq sequencer using a 2 × 250-bp paired-end chemistry (600 cycles) (Illumina) (9 – 11). The quality of the raw short reads (FASTAQ files) was checked using FastQC tool version 0.11.9 (12), and reads (*N* = 26,377,800 bp) were trimmed using Trimmomatic v0.39 to remove Illumina adapters, known as Illumina artifacts and PhiX reads (13). Genome sequences were assembled using SPAdes v.3.15.5 (14), and quality of the assembled genome was checked using QUAST v.5.0.2 (15). The genome sequences of the MBR-MFRK-23 isolate was annotated using the NCBI Prokaryotic Genome Annotation Pipeline v6.4 (16), while BacWGSTdb 2.0 (17) was used for sequence typing. ResFinder 4.0 (18), PlasmidFinder (19), VFDB (20), and RAST FIGfams v.70 (21) databases were used to predict antimicrobial resistance genes (ARGs), plasmids, virulence factors, and metabolic functions, respectively, in the assembled genome. Default parameters were used for all software unless otherwise stated (9, 10).

The features of the draft genome are presented in Table 1. The MBR-MFRK-23 strain was typed as *S. enterica* sequence type 19, according to seven-gene core-genome multilocus sequence typing (MLST) (e.g., *aro*C, *dna*N, *hem*D, *his*D, *pur*E, *suc*A, and *thr*A). We predicted 370 metabolic features, 100 ARGs, and 128 virulent genes in the draft genome. Four plasmid replicons, such as Col156 (3,981 bp), ColpVC (2,173 bp), IncFIB(S) (48,081 bp), and IncFII(S) (11,541 bp), were predicted in this genome by PlasmidFinder, an *in silico* plasmid detection and typing tool (with >98% identity and 80% coverage).

**TABLE 1:** Genomic features of the *S.* Typhimurium strain MBR-MFRK-23

Isolate	Genome coverage (×)	Genome size (bp)	No. of contigs	*N*50 value (bp)	GC content (%)	No. of protein-coding sequences	No. of RNA gene
MBR-MFRK-23	50.5	4,964,854	50	225,558	52.1	5,085	77

## Data Availability

The whole-genome shotgun project of the *S. enterica* serovar Typhimurium strain MBR-MFRK-23 has been deposited at NCBI/GenBank under the accession number JARQGX000000000, and the raw reads were deposited under SRA accession number SRR24966878 (BioProject accession number PRJNA948197). The version described in this paper is version JARQGX000000000.1.
